# Hotspots and future directions in rheumatoid arthritis-related cardiovascular disease: A scientometric and visualization study from 2001 to 2021 based on Web of Science

**DOI:** 10.3389/fmed.2022.931626

**Published:** 2022-07-29

**Authors:** Pengfei Wen, Pan Luo, Binfei Zhang, Yakang Wang, Linjie Hao, Jun Wang, Jianbin Guo, Rui Liu, Yumin Zhang, Juan Chen

**Affiliations:** Department of Joint Surgery, Honghui Hospital, Xi’an Jiaotong University, Shaanxi, China

**Keywords:** scientometric analysis, rheumatoid arthritis, knowledge structure, hotspots, research trends, cardiovascular disease

## Abstract

**Background:**

The morbidity and mortality of cardiovascular diseases (CVD) in patients with rheumatoid arthritis (RA) is significantly higher than those in the general population, leading to RA-related CVD has attracted broad attention and numerous articles have been published. However, no study has systematically examined this area from a scientometric perspective. This study aimed to visualize the knowledge structure and identify emerging research trends and potential hotspots in this field.

**Materials and methods:**

Articles and reviews on RA-CVD published from 2001 to 2021 were extracted from the Web of Science Core Collection database. CiteSpace and VOSviewer software were used to visualize the knowledge network of countries, institutions, authors, references and keywords in this field. SPSS and Microsoft Excel software were used for curve fitting and correlation analysis.

**Results:**

A total of 2,618 articles and reviews were included. The number of publications about RA-related CVD significantly increased yearly. Publications were mainly concentrated in North America, Europe and East Asia. The United States contributed most with 699 publications, followed by the United Kingdom and Italy. Gross Domestic Product was an important factor affecting scientific output. University of Manchester and Professor Kitas George D. were the most prolific institutions and influential authors, respectively. Journal of Rheumatology was the most productive journal for RA-related CVD research. The research hotspots switched in the order of clinical features (cardiovascular events), mechanism exploration, anti-tumor necrosis factor therapy, risk factors, and antirheumatic drug safety, which can be observed from the keyword analysis and co-cited reference cluster analysis.

**Conclusions:**

This study found that research on RA-related CVD is flourishing. The safety and cardiovascular pharmacological mechanisms of anti-rheumatoid drugs, especially targeted synthetic DMARDs, would be the focus of current research and developmental trends in future research.

## Introduction

Rheumatoid arthritis (RA) is a systemic autoimmune disease with an approximately 0.5–1% prevalence worldwide (1). Its main extra-articular feature is the excessive morbidity and mortality of RA-related cardiovascular disease (CVD). The risk of CVD events in RA patients is 3.17 times higher than in the non-RA population (2). As reported, the risk of myocardial infarction, atrial fibrillation and stroke is increased by 68, 41, and 32% in RA patients, respectively (3, 4). Moreover, patients with RA have almost twice the risk of developing congestive heart failure (5). The occurrence of RA-related CVD is mostly considered to be the result of immune system dysregulation and systemic inflammation (1, 6), while it has been suggested to be associated with the side effects of therapeutic agents, such as non-steroidal anti-inflammatory drugs (NSAIDs) and glucocorticoids (7, 8). In addition, CVD is the leading cause of death in RA patients, accounting for up to 39.6% of RA patients, which is 50% higher than the general population (9, 10).

Given the above aspects, RA-related CVD has received increasing attention from scholars. However, there are still some controversies surrounding RA- related CVD, such as CVD risk factors, prevention drugs, and optimal treatment protocols (1). Motivated by these controversies, a large number of studies on RA-related CVD have been published. The rapidly growing number of publications makes it increasingly difficult for researchers to fully understand, evaluate and identify the most relevant and valuable information in the field, especially for new investigators. Therefore, it is necessary to provide a macroscopic description of research trends, hotspots, and high-impact articles, institutions and authors in this field.

New researchers can benefit from an overview of the knowledge structure and current hotspots in a given field (11–13). Scientometric analysis has become an increasingly popular method to obtain the aforementioned parameters. This method can quantitatively and qualitatively analyze the scientific achievements and the current status of a certain research field. It has been widely used in medical fields such as oncology (14, 15), orthopedics (16–18), COVID-19 (19), urology (20), and rheumatology (12, 13). Taking RA and CVD as examples, Wu et al. conducted scientometric research to discuss publication status and research hotspots in the RA-related osteoporosis field (12). Furthermore, some scholars have investigated and mapped the overall knowledge structure of the CVD field through scientometrics (21). Nevertheless, to our knowledge, no attempt has been made so far to analyze the field of RA-related CVD, although there have been some scientometric studies on RA or CVD. Hence, this study aimed to comprehensively analyze the scientific publications on RA-related CVD research from 2001 to 2021, so as to determine the current research status and knowledge structure and to predict future development prospects.

## Materials and methods

### Data sources and search strategies

**FIGURE 1:**
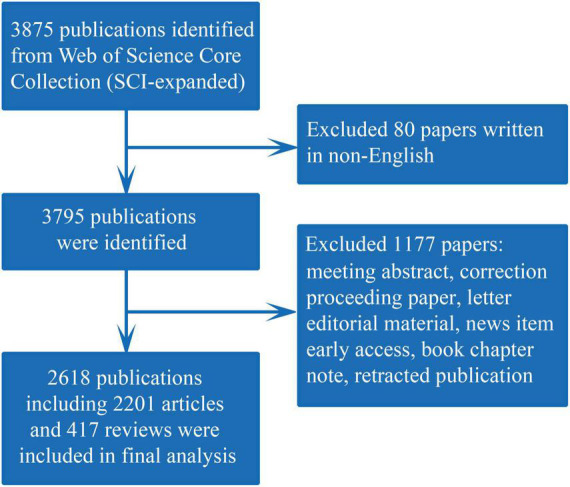
Flowchart of the identification of relevant articles.

Web of Science (WoS) contains more than 12,000 international academic journals, which is one of the most comprehensive and authoritative database platforms for accessing global academic information and is widely used for scientometric analysis (12). In our study, all literature was retrieved and downloaded from the Science Citation Index Expanded of WoS Core Collection (WoSCC) database on March 8, 2022, to avoid bias caused by database updates. Since RA was the main subject of this study, a search was performed for terms related to RA according to the title (TI) to obtain more precise results. The specific search strategy was as follows: TI = (Rheumatoid arthritis) AND (TS = heart or TS = cardiovascular), and the time span was set to 2001-2021 by referring to previous literature (11, 12). A total of 3,875 publications were retrieved, of which 1,257 invalid records were excluded, including essays, edited materials, corrections, meeting summaries, letters, early visits, retracted publications and non-English literature works. Finally, 2,618 valid publications were obtained as the final dataset for further analysis (Figure 1).

### Data extraction

Data extraction was performed by two independent researchers to ensure the accuracy and reliability of the results. Any disagreements between the two researchers were discussed until a consensus was reached. The extracted data included authors, titles, publication years, citation times, countries, institutions, journals, highly cited articles, references, and keywords. The Hirsch index (H-index) was obtained by WoS. In addition, taking into account the differences in economic and demographic conditions in different countries, several ratio indexes were introduced, including the number of papers per million people and the number of papers per trillion Gross Domestic Product (GDP) (12).

### Data analysis

Statistical analysis was performed using IMB SPSS 22.0 (IBM Corp., Armonk, NY, United States), and Microsoft Excel 2016 (Microsoft Corp., Redmond, WA, United States). Statistical plots were drawn using OriginPro 9.1 (OriginLab Corp., Northampton, MA, United States). Categorical data were expressed as counts (percentages). The correlation strength between continuous variables was assessed by Pearson’s correlation coefficient. *P* < 0.05 was defined as statistically significant.

Visualization software including VOSviewer, CiteSpace and Scimago Graphica were used in the scientometrics analysis. Scimago Graphica software was used to map the global distribution of national publications and the cooperation network of countries. VOSviewer software was used to visualize the co-authorship analyzes of institutions and authors, as well as the co-occurrence analysis of keywords. CiteSpace was mainly used for reference co-citation analysis, keyword burst detection, and drawing timeline views of reference clusters.

## Results

### Trends in publications and citations

**FIGURE 2:**
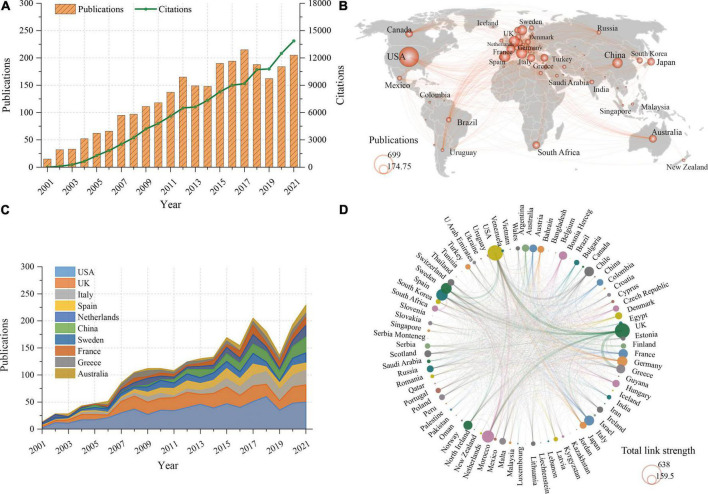
**(A)** Trends in annual citations and publications about rheumatoid arthritis-related cardiovascular disease from 2001 to 2021. **(B)** World map showing the contribution of each country. **(C)** Trends in annual publication count in the top 10 productive countries. **(D)** Cooperation network among countries. The thickness of lines between two countries indicates the strength of cooperation.

After the literature screening, a total of 2,618 publications were included in the final analysis, including 2201 original articles and 467 reviews. The detailed distribution of annual publications for RA-related CVD research was shown in Figure 2A. It can be seen that, despite fluctuating declines at certain time points, the number of annual publications on RA-related CVD showed an overall upward trend and reached its peak in 2017 with 215 articles. When it comes to the number of citations, the cumulative total number of citations for these publications was 120,880 (90,066 after removing self-citations), and the average number of citations per publication was 46.17. As can be seen from the distribution of annual citations (Figure 2A), it showed a linear growth trend (R^2^ = 0.9849).

### Global research status and knowledge structure

#### Analysis of countries

From the world map of country contributions (Figure 2B), it can be observed that the vast majority of works were published by researchers from North America, Europe and East Asia. Specifically, as shown in Table 1, the United States has published the most papers in this field, with 699 (26.7%) papers, followed by the United Kingdom (United Kingdom), Italy and Spain, while other countries have published less than 200 papers. Moreover, after adjusting by population size and GDP, Greece occupied the first position with 534.9 papers per trillion GDP and Sweden with 1574.4 papers per million people. In addition, the study found no significant correlation between the number of publications and demographic data (*r* = 0.221, *p* = 0.077), while there was a high positive correlation between the number of publications and GDP (*r* = 0.797, *p* < 0.001).

**TABLE 1:** Top 10 countries with the most publications.

Rank	Country	No. of papers	Total citation	Average citation	H-index	No. of papers per trillion GDP	No. of papers per million people
1	United States	699	51,107	73.11	105	33.36	212.15
2	United Kingdom	349	26,032	74.59	81	126.46	519.23
3	Italy	208	9,065	43.58	42	110.13	349.26
4	Spain	203	11,837	58.31	48	158.41	428.71
5	Netherlands	198	16,246	82.05	55	216.66	1,135.25
6	China	177	5,047	28.51	29	12.02	12.54
7	Sweden	163	12,640	77.55	50	301.17	1,574.36
8	France	128	9,371	73.21	40	48.66	189.93
9	Greece	101	5,261	52.09	35	534.86	942.56
10	Australia	98	6,280	64.08	34	73.80	381.52

GDP, Gross Domestic Product; The Demographic and GDP data were obtained from the World Bank official website.

As shown in Figure 2C, the United States and the United Kingdom always dominated the number of papers in this field. Of note, Italy and China experienced rapid growth since 2011. Figure 2D showed the international cooperation among the different countries. The thickness of lines between two countries indicates the strength of cooperation. It can be seen that the United Kingdom had the closest cooperation with the United States, Morocco and Greece. In general, most collaborations were mainly limited to Europe, North America and East Asian countries.

#### Analysis of institutions

**FIGURE 3:**
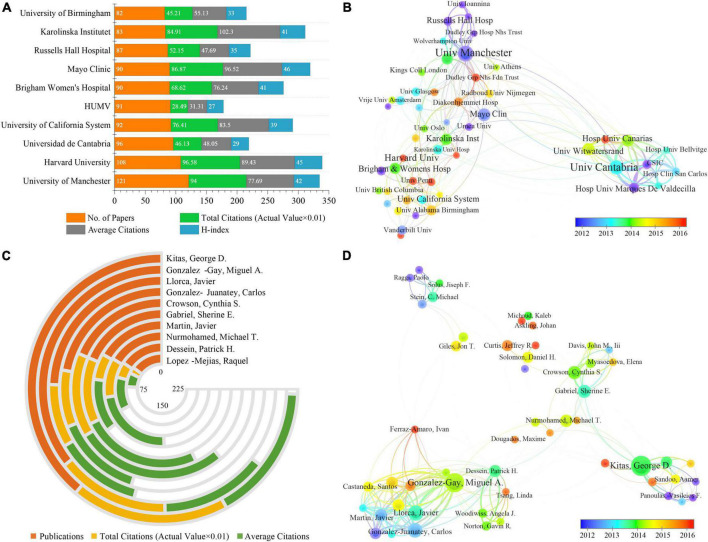
**(A)** The publication counts, citations, and H-index of the top 10 prolific institutions. HUMV stands for Hospital Universitario Marqués de Valdecilla. **(B)** Cooperation network among institutions. **(C)** The publication counts and citations of the top 10 most productive authors. **(D)** Cooperation network among authors. The nodes in the graph represent institutions or authors, and lines between the nodes represent collaborative relationships. Nodes are marked with different colors depending on the average appearing year. The thickness of lines between two institutions or authors indicates the strength of cooperation, while the area of nodes represents the number of publications.

It was roughly estimated that more than 2,000 institutions contributed to this field. Figure 3A showed the top 10 most productive institutions. Four of these are from the United States, three from the United Kingdom, two from Spain and one from Sweden. Specifically, the University of Manchester in the United Kingdom ranked first with 121 articles, followed by Harvard University^1^ in the United States. The Karolinska Institutet in Sweden had the highest average citation of 102.3. Mayo Clinic had the highest H-index of 46.

Figure 3B highlighted the close and complex cooperation among different institutions. It can be seen that inter-institutional collaborations were scattered within high-income countries such as North American and European countries. The cooperations centered on the Universidad de Cantabria and the University of Manchester were the most frequent, indicating a strong influence of these institutions. In addition, several institutions marked in red, such as Harvard University, University of Pennsylvania, Hospital Universitario de Canarias and Diakonhjemmet Hospital, were relatively new participants in the RA-related CVD research.

#### Analysis of journals

During the last two decades, a total of 514 academic journals published papers on RA-related CVD. Table 2 summarized the characteristics of the top 10 most prolific journals. Of these, the Journal of Rheumatology (169, 6.46%) published the most, followed by Annals of the Rheumatic Diseases (166, 6.34%) and Arthritis & Rheumatology (128, 4.89%). As far as the research fields of these journals were concerned, 70% of these journals were classified as rheumatology. In terms of publishers, four of these journals were from the United Kingdom, three from the United States, and the others from Canada, Germany and Italy, respectively. Notably, most of these active journals were located in Europe and North America.

**TABLE 2:** The top 10 journals contributing to publications.

Rank	Journal	No. of papers	Percentage (%)
1	Journal of Rheumatology	169	6.46
2	Annals of the Rheumatic Diseases	166	6.34
3	Arthritis & Rheumatology^†^	128	4.89
4	Arthritis Research & Therapy	125	4.78
5	Clinical Rheumatology	121	4.62
6	Rheumatology	118	4.51
7	Clinical and Experimental Rheumatology	113	4.32
8	Arthritis Care & Research	88	3.36
9	Rheumatology International	83	3.17
10	Plos One	58	2.90

^†^Arthritis and Rheumatism relaunched as Arthritis & Rheumatology after 2015. The data from these journals were merged.

### Overview of landmark articles and authors

#### Analysis of authors

In terms of the top 10 prolific authors (Figure 3C and Table 3), Kitas George D. from Russell Hall Hospital ranked first, followed by Gonzalez-Gay Miguel A. and Llorca Javier. In addition, they were also the top three authors with the highest H-index. Gonzalez-Gay Miguel A. and Llorca Javier were from the same institution, the Universidad de Cantabria. Of note, half of the top 10 authors were from Spanish institutions.

**TABLE 3:** Top 10 most prolific authors on rheumatoid arthritis-related cardiovascular disease.

Rank	Author	No. of papers	Total citations	Average citations	Institution	Country
1	Kitas George D.	115	5,583	48.55	Russells Hall Hospital	United Kingdom
2	Gonzalez-Gay Miguel A.	105	4,366	41.58	Universidad de Cantabria	Spain
3	Llorca Javier	72	2,669	37.07	Universidad de Cantabria	Spain
4	Gonzalez-Juanatey Carlos	65	2,680	41.23	HULA	Spain
5	Crowson Cynthia S.	55	4,014	72.98	Mayo Clinic	United States
6	Gabriel Sherine E.	55	4,452	80.95	Mayo Clinic	United States
7	Martin Javier	49	1,817	37.08	CSIC	Spain
8	Nurmohamed Michael T.	48	3,284	68.42	Vrije Universiteit Amsterdam	Netherlands
9	Dessein Patrick H.	46	1,590	34.57	Vrije Universiteit Brussel	Belgium
10	Lopez-Mejias Raquel	44	828	18.82	IDIVAL	Spain

CSIC, Consejo Superior de Investigaciones Científicas; IDIVAL, Instituto de Investigación Sanitaria Valdecilla; HULA, Lucus Augusti University Hospital.

A visualization of the author’s co-authorship analysis was generated by VOSviewer software (Figure 3D). It can be seen that there were several research clusters, each of which was radiated by one or two core authors such as Kitas George D, Gonzalez-Gay Miguel A. and Crowson Cynthia S. Authors at the connection points between different clusters played a bridging role in institutional cooperations, such as Ferraz-Amaro Ivan, Kitas George D and Gabriel Sherine E. In addition, several authors marked in red, such as Ferraz-Amaro Iva and Curtis Jeffrey R., were new active participants in RA-related CVD research.

#### Analysis of highly cited literature

**FIGURE 4:**
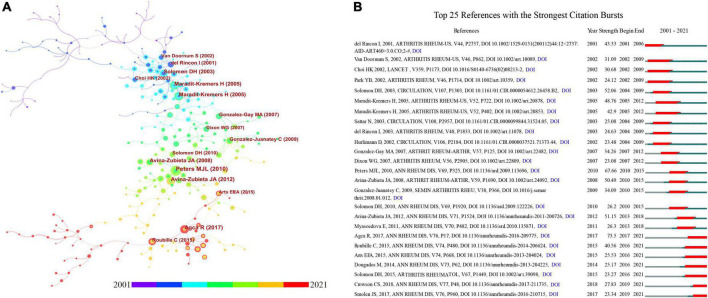
**(A)** The co-cited reference knowledge map. **(B)** The top 25 references with the strongest citation bursts.

Table 4 showed the details of the top 10 most cited papers on RA-related CVD with citations ranging from 512 to 1,058. Most of the studies were published in rheumatology journals such as Annals of the Rheumatic Diseases, Journal of Rheumatology, and Arthritis & Rheumatology. Seven of these articles were original articles and three were systematic reviews. Figure 4A illustrated a knowledge map of highly co-cited references. Figure 4B specifically showed the top 25 references with the strongest citation bursts, in which the blue line represented the time interval, and the red part indicated the period when the reference burst occurred. Among these references, the reference with the strongest citation burst value was written by Agca, R et al. (22).

**TABLE 4:** Top 10 high-cited articles on rheumatoid arthritis-related cardiovascular disease.

Authors	Year	Article title	Journal	Total citations
del Rincon, I, et al. (2)	2001	High incidence of cardiovascular events in a rheumatoid arthritis cohort not explained by traditional cardiac risk factors	Arthritis and Rheumatism^†^	1058
Solomon, DH, et al. (25)	2003	Cardiovascular morbidity and mortality in women diagnosed with rheumatoid arthritis	Circulation	939
Peters, MJL, et al. (26)	2010	EULAR evidence-based recommendations for cardiovascular risk management in patients with rheumatoid arthritis and other forms of inflammatory arthritis	Annals of The Rheumatic Diseases	934
Avina-Zubieta, JA, et al. (10)	2008	Risk of Cardiovascular Mortality in Patients With Rheumatoid Arthritis: A Meta-Analysis of Observational Studies	Arthritis and Rheumatism^†^	909
Maradit-Kremers, H, et al. (53)	2005	Cardiovascular Death in Rheumatoid Arthritis A Population-Based Study	Arthritis and Rheumatism^†^	711
Sattar, N, et al. (6)	2003	Explaining how high-grade systemic inflammation accelerates vascular risk in rheumatoid arthritis	Circulation	699
Maradit-Kremers, H, et al. (53)	2005	Increased unrecognized coronary heart disease and sudden deaths in rheumatoid arthritis - A population-based cohort study	Arthritis and Rheumatism^†^	691
Agca, R, et al. (22)	2017	EULAR recommendations for cardiovascular disease risk management in patients with rheumatoid arthritis and other forms of inflammatory joint disorders: 2015/2016 update	Annals of The Rheumatic Diseases	583
Avina-Zubieta, JA, et al. (4)	2012	Risk of incident cardiovascular events in patients with rheumatoid arthritis: a meta-analysis of observational studies	Annals of The Rheumatic Diseases	523
Han, CL, et al. (54)	2006	Cardiovascular disease and risk factors in patients with rheumatoid arthritis, psoriatic arthritis, and ankylosing spondylitis	Journal of Rheumatology	512

^†^Arthritis and Rheumatism relaunched as Arthritis & Rheumatology after 2015.

### Overview of research hotspots and frontiers

#### Cluster analysis of co-cited references

**FIGURE 5:**
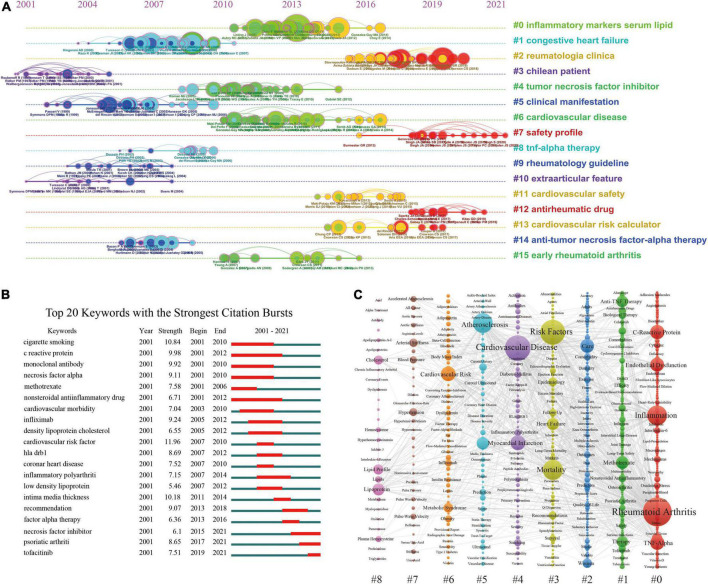
**(A)** The timeline graph of co-cited reference clusters. **(B)** The top 20 keywords with the strongest citation bursts. **(C)** The co-occurrence view of the keywords, in which each column represents a cluster of studies generated by VOSviewer.

The references in the co-cited network (Figure 4A) were divided into 16 different clusters by cluster analysis shown in the timeline view (Figure 5A). These clusters were labeled by extricating terms from the titles of cited publications. As can be seen in Figure 5A, inflammatory markers serum lipid was the largest cluster (#0), followed by congestive heart failure (#1) and reumatologia clinica (#2). Moreover, the evolution characteristics of each cluster can be known from this timeline view. The focuses of RA-related CVD research shifted in the order of clinical features (#1, # 3, # 5, # 10), mechanism exploration (#0, #6, #15), anti-tumor necrosis factor therapy (# 4, #8, #14), CVD risk factors (#11, #13) and antirheumatic drug safety (#7, #12).

#### Analysis of keywords

In the keyword analysis, the meaningless keywords were excluded and the same meaning keywords were merged. Figure 5B listed the top 20 keywords with the strongest citation burst. It can be known that tofacitinib was the strongest burst keyword in recent 2 years. Figure 5C showed a visualization of keywords that co-occurred more than 5 times in the RA-CVD research. The size of the node was proportional to the frequency of keyword occurrences, and a thicker line between two nodes was associated with a higher frequency of their co-occurrence (12, 15). A total of 628 keywords were divided into 9 groups. According to the keywords in the groups, the groups can be summarized as follows: #0 inflammatory mechanism, #1 treatment, #2 care, #3 mortality, #4 cardiovascular disease, #5 atherosclerosis, #6 risk factor, #7 blood pressure, and #8 lipid.

## Discussion

This study provided a scientometric analysis of RA-related CVD research from 2001 to 2021 to find milestone achievements and predict new research hotspots. It can also help beginners intuitively and systematically understand the development process and trends in this field. This study found that the number of publications and citations of RA-related CVD have been numerous and increasing in the past two decades (Figure 2A). Accordingly, we believe that RA-related CVD has attracted a lot and growing attention from scholars, and it is an important research field all over the world.

From the perspective of national contribution, the United States ranked first in the world in terms of publications, citations and H-index, indicating its dominant influence (Figures 2B and Table 1). Four of the top 10 prolific institutions were located in the United States, three in the United Kingdom, two in Spain, and one in Sweden (Figure 3A). The influence and contribution of scientific institutions fundamentally represent a country or region. In summary, most of the works were produced in high-income countries, such as those in North America, Europe, and East Asia. This study found a significant positive correlation between country output and GDP, indicating that economic power was an important factor affecting scientific activities. Countries with high GDP may allot substantial investments in scientific investigations and foster a large number of senior researchers (12, 23). Co-authorship analysis, a popular method, establishes similarity relationships between individuals or groups through the number of co-authored publications, from which the status of collaboration between institutions or individuals can be understood (12). Figure 3B revealed that most cooperative institutions were confined to internal contacts, and cross-border cooperation was greatly reduced. Therefore, it is strongly recommended that the academic barriers should be removed and collaboration and communication should be improved between institutions to promote RA-related CVD research and development (12, 24).

Publishing research results in international peer-reviewed journals is an important part of establishing effective scientific communication (12). The analysis of the distribution of journal sources could help researchers to quickly find the most suitable journals for their works. As can be seen from Table 2, the Journal of Rheumatology published the most in this field, followed by Annals of the Rheumatic Diseases and Arthritis & Rheumatology, representing their high reputation and authority. The number of scientific articles published by an author can be a good representation of his research activities and contributions to the field. The data from Kitas George D. (Russells Hall Hospital, United Kingdom) were particularly attractive in terms of publications and citations (Table 3 and Figure 3C). He has contributed to 115 publications on RA-related CVD research with a total of 5,583 citations.

By analyzing Table 4 and Figure 4, it can be found that the top 10 cited publications (Table 4) were all included in the references with the strongest citation bursts (Figure 4). In other words, literature with high citations can be considered as the most valuable and influential research in the field, so new researchers can read these papers before conducting further research. In 2001, del Rincon I, et al. first reported that RA was an independent risk factor for CVD and the CVD risk in RA patients was 3.17 times higher than that in the general population (2). Subsequently, several relevant studies and meta-analyzes have confirmed this view (3, 4, 10, 25). In 2003, a review published in Circulation identified that the systemic inflammatory response in RA was critical for accelerating atherosclerosis, which operates *via* accentuation of established and novel risk factor pathways. By implication, long-term suppression of the systemic inflammatory response in RA should effectively reduce the risk of CVD (6). In 2009, the European League Against Rheumatism (EULAR) taskforce introduced recommendations for screening, identification of CVD risk factors and CVD risk management in patients with RA based on expert opinion (26). Subsequently, the guide was updated based on extensive new research evidence and quickly gained a large number of citations and wider acceptance by researchers (22). This article also had the strongest citation burst of 73.5, and its burst period has lasted until now (Figure 4B).

Cluster analysis of co-cited references and co-occurrence keywords has been proved to reflect the research topics and hotspots in the field (27, 28). In this study, co-cited references were divided into 16 clusters by CiteSpace software (Figure 5A), while keywords were divided into 9 clusters by VOSviewer (Figure 5C). Cluster numbers were sorted according to the size of the clusters, which indicates that larger clusters mean more relevant studies or keywords within them. In the keyword cluster #0, except for rheumatoid arthritis, other frequently occurring keywords such as inflammation, C-reactive protein, tumor necrosis factor (TNF) alpha, interleukin-6 (IL-6), interleukin-1 (IL-1), oxidative stress, endothelial dysfunction, etc., indicated that this cluster mainly focused on the molecular mechanism and signal pathway of RA-related CVD. It is generally accepted that high levels of pro-inflammatory cytokines and immune system dysregulation play a key role in the progression of RA-related CVD *via* accelerating the atherosclerosis process (7, 29, 30).

It is worth discussing that in keyword cluster #1, the common keywords were DMARD, anti-TNF therapy, biological therapy, methotrexate, tofacitinib, NSAIDs and glucocorticoids, indicating that this cluster mainly focused on the research of anti-rheumatoid drugs. Although these drugs have shown great improvements in RA control in the last decades, the incidence of RA-related CVD remains high (4, 31, 32). The evidence demonstrated that some drugs could also directly or indirectly promote atherosclerosis, thus increasing the risk of CVD, such as NSAIDs and glucocorticoids (8, 33). The increased risk of cardiovascular events and stroke with NSAIDs may be through their effects on blood pressure and coagulation as well as oxidative stress (34). Glucocorticoids have adverse effects on lipid metabolism and glucose homeostasis and can reduce the availability of nitric oxide thereby promoting the formation of oxidative stressors, thus at least partially explaining their detrimental cardiovascular effects (35). Therefore, the use of these drugs must be weighed against their positive effects on RA and their deleterious effects on cardiac metabolism.

In contrast, increasing evidence suggested that methotrexate, TNF inhibitor and IL-6 inhibitor (tocilizumab) could effectively inhibit inflammation and reduce the risk of RA-related CVD (36–40). Broad inhibition of several immune pathways may explain the protective effect of methotrexate on cardiovascular damage in RA. For example, studies have highlighted various mechanisms by which methotrexate has anti-inflammatory and cardioprotective effects, including by promoting reverse cholesterol transport (41), increasing the ability of high-density lipoprotein (HDL) to promote cholesterol efflux (opposing the differentiation and activation of foam cells) and reducing the expression of adhesion molecules on endothelial cells (inhibiting the recruitment of pro-inflammatory cells, such as T cells and monocytes) (29, 42). TNF inhibitors and IL-6 inhibitors were considered to have similar cardioprotective mechanisms (43). However, for other conventional synthetic (sulfasalazine, hydroxychloroquine, cyclosporine, leflunomide), biologic (rituximab, abatacept, IL-1 inhibitors), and targeted synthetic (JAK inhibitors) DMARDs, it remains uncertain whether these drugs will affect cardiovascular risk in RA patients due to limited evidence (1, 7, 44).

Timeline plots of clusters can track the evolution of research hotspots and predict the research trends in the coming years (28). In addition, the burst detection algorithm developed by Kleinberg can capture the sharp increase in keyword popularity over a specific period, which can be used as an effective way to identify concepts or topics discussed actively during this period (45). Figures 5A,B revealed a shift in the research foci of RA-CVD in the order of clinical features, mechanism exploration, risk factors and antirheumatic drug safety. In recent years, the research hotspot may be on the safety and mechanism of anti-rheumatoid drugs which is predicted to receive continuous attention in the future as well. Taking tofacitinib as an example, Janus Kinase (JAK) inhibitor is a targeted synthetic DMARDs that disturbs cytokine signaling by interfering with the JAK-STAT signaling pathway (46). Studies suggested that this drug modifies the function and composition of HDL fractions, transforming them from pro-inflammatory to anti-inflammatory particles (47, 48). In addition, the regulation of some JAK-dependent cytokines might be its potential mechanism, as these cytokines are associated with the pathogenesis of atherosclerosis (49, 50). Its benefits for RA treatment have been demonstrated by several clinical and laboratory studies (50–52). Nevertheless, the scientific evidence on the safety of these drugs is still limited, especially no long-term studies have been conducted so far.

Taken together, this study has described the research status, hotspots and further trends in the RA-related CVD. Researchers, especially newcomers, could benefit from this study as they could clearly understand the basic knowledge structure of the field, including countries, institutions, authors and journals, and be inspired by the research hotspots and frontiers. We have to acknowledge that this study has some inherent limitations in scientometrics. First, the databases are constantly updated and only the WoSCC database was analyzed in this study. Some articles published in other databases may be omitted. However, as described in previous studies, WoSCC was the most commonly used and sufficiently large database for scientometric analysis to reflect the current status in a given field (12, 16, 17). Second, only English publications were selected and non-English publications were ignored, which implies that the value of non-English publications may be underestimated. Third, the impact of recently published high-quality articles may also be underestimated because they may not have sufficient time to accumulate enough citations. A follow-up study could be conducted in the future to evaluate the influence of these articles in the field.

## Conclusion

To our knowledge, this was the first study to conduct a comprehensive scientometric analysis of global publications on RA-related CVD from 2001 to 2021. Our findings suggested that RA-related CVD has attracted considerable and growing attention from scholars. Up to now, North American and European countries have been leaders in this field, which is inseparable from adequate funding sources. University of Manchester and Professor Kitas George D. were the most prolific institutions and influential authors, respectively. Journal of Rheumatology and Annals of the Rheumatic Diseases were the most prolific and influential journals in RA-related CVD research, with the largest number of publications and citations, respectively. Cardiovascular events, mechanism exploration, risk factors and antirheumatic drug safety were the hotspots in this field. Of note, the research foci have shifted with time, and the safety and cardiovascular pharmacological mechanism of anti-rheumatoid drugs, especially targeted synthetic DMARDs, are considered to be important research directions in the future. Long-term, robust and well-designed clinical trials deserve further attention.

## Data availability statement

The original contributions presented in the study are included in the article/supplementary material, further inquiries can be directed to the corresponding authors.

## Author contributions

PW and PL: writing-original draft. PW and YZ: conceptualization, project administration, and writing-review and editing. PL, BZ, YW, and JC: data curation and methodology. LH, JW, and JC: formal analysis and validation. PW, RL, and JG: visualization and software. All authors contributed to the article and approved the submitted version.

## Footnotes

1
https://data.worldbank.org.cn/
